# Predation risk is a function of alternative prey availability rather than predator abundance in a tropical savanna woodland ecosystem

**DOI:** 10.1038/s41598-019-44159-6

**Published:** 2019-05-22

**Authors:** Eric J. Nordberg, Lin Schwarzkopf

**Affiliations:** College of Science and Engineering, James Cook University, Townsville, QLD 4811 Australia

**Keywords:** Food webs, Food webs

## Abstract

Typically, factors influencing predation risk are viewed only from the perspective of predators or prey populations but few studies have examined predation risk in the context of a food web. We tested two competing hypotheses regarding predation: (1) predation risk is dependent on predator density; and (2) predation risk is dependent on the availability of alternative prey sources. We use an empirical, multi-level, tropical food web (birds–lizards–invertebrates) and a mensurative experiment (seasonal fluctuations in abundance and artificial lizards to estimate predation risk) to test these hypotheses. Birds were responsible for the majority of attacks on artificial lizards and were more abundant in the wet season. Artificial lizards were attacked more frequently in the dry than the wet season despite a greater abundance of birds in the wet season. Lizard and invertebrate (alternative prey) abundances showed opposing trends; lizards were more abundant in the dry while invertebrates were more abundant in the wet season. Predatory birds attacked fewer lizards when invertebrate prey abundance was highest, and switched to lizard prey when invertebrate abundance reduced, and lizard abundance was greatest. Our study suggests predation risk is not predator density-dependent, but rather dependent on the abundance of invertebrate prey, supporting the alternative prey hypothesis.

## Introduction

The complex interactions between predators and their prey are instrumental in shaping the composition and structure of animal assemblages^1^. A wide range of theory has been developed to predict the responses of predators to prey, both at the level of the population, and of individuals^2,3^. Although it is simpler to conceptualize predator–prey systems using mathematical modelling, interactions in nature may behave in ways not captured in models, and therefore empirical studies of such interactions are extremely valuable to tease apart the factors driving assemblage dynamics. Predation risk is, for example, a critical factor influencing the evolution of morphology, ecology, and life-history traits, of both prey and predators alike^4–6^. However, predation risk is notoriously difficult to measure in natural systems, and is typically inferred using indirect measures such as changes in habitat use or foraging behaviour of prey^5,7,8^ rather than empirical measures of predation risk or direct mortality^9–11^. Direct, empirical measures of predation risk in the field are useful to support the conclusions from such inferences.

Multiple factors can affect predation risk, including the abundance and density of predators. Predation risk is not, however, solely dependent on the abundance, or density of predators, as the abundance of prey populations are also important factors determining predation in multiple-prey systems^12–14^. Predators that change their prey preference, or exhibit prey-switching, consume prey disproportionally less than their availability when prey are rare or at low densities, and disproportionally more than their availability when prey are at high prey densities^15^. Prey-switching can be beneficial to predators, ensuring maximum food intake per unit effort, and maximizing foraging efficiency^16–18^. In addition, prey-switching can be protective for prey abundance because as a primary prey group becomes depleted, predators switch to alternative prey sources, allowing depleted primary prey populations to recover^19^. Further, prey-switching promotes prey species diversity, coexistence with predators, and may stabilize wildlife populations^20^.

Changes in predation risk in response to predators may, thus, be explained by one of two hypotheses: the shared predation hypothesis, which suggests that all prey groups are at high risk when predator populations are large^12,21^; or the alternative prey hypothesis, which states that predators switch prey groups when primary prey densities drop below the density of alternative prey^22,23^. Typically, factors influencing predation risk are examined either from the point of view of individual predator populations (i.e., examining functional and numerical responses)^24,25^ or of prey populations (i.e, focusing on direct or non-consumptive [indirect] behavioural effects of predators on prey)^5,7,8^. Few studies have directly quantified predation risk in relation to predator abundance or density; instead many assume that more predators lead to higher predation risk (but see^26–29^). If the extent of predation risk is not measured directly, but instead only inferred from the abundance of predators, or the behaviour of prey, it is not possible to distinguish which of these factors is more important in driving predation risk. Understanding the relative importance of these processes in determining predation risk is critical to translating the predictions of predator–prey models to explain the outcomes of real population dynamics and community processes.

Few studies incorporate both predator and prey densities and their impacts on direct measures of predation risk (but see^30–32^). We used a mensurative experiment in a model system with changing predator and prey abundances, to directly quantify predation risk and examine the relative role of predator population size and prey availability in determining predation risk. The primary objective was to empirically test two hypotheses regarding predation risk: (1) is predation risk dependent on predator densities, i.e., does predation risk increase with an increase in predator abundance; or (2) is predation risk inversely dependent on the abundance of alternative prey (alternative prey hypothesis)? In this study, we used a multi-level tropical food web with small lizards and invertebrates as prey groups, and avian and large invertebrates (e.g., huntsman spiders) as predators. We used artificial lizard models to estimate predation risk on lizards. We simultaneously quantified the abundance of predators and alternative prey to test if predation risk was proportional to predator abundance or if predation risk to lizards was more closely related to the abundance of alternative prey.

## Results

### Artificial lizard predation

**Figure 1:**
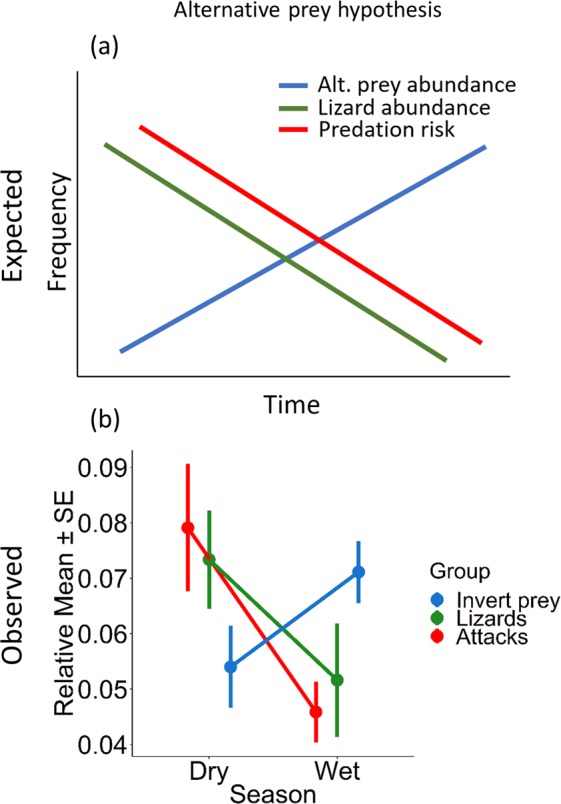
Expected (**a**) and observed (**b**) results for the alternative prey hypothesis, i.e., predation risk is inversely dependent on the abundance of alternative prey. The observed values for the alternative prey hypothesis (**b**) indicate that predation risk on lizard models (Attacks; red) was inversely related to the relative mean abundance of invertebrate (alternative) prey (Invert. Prey; blue), and proportional to the relative mean abundance of living lizards (Lizards; green), showing support for the alternative prey hypothesis. The relative means represent the mean ± SE of the responses (attacks on artificial lizards models, invertebrate prey abundance, and *Gehyra dubia* abundance) as a proportional representation summarized by season to scale all the data from 0–1, and compare the responses.

Predators attacked control models significantly less than lizard-shaped models during the wet season (when both control and artificial lizards were present) (*t* = −3.394, df = 13, *P* = 0.004; Table 1). Our top models included *invertebrate abundance*, *season*, *habitat type*, and *microhabitat* as the best predictors of artificial lizard attack rates (Table 2). There were more attacks on artificial lizards when invertebrate prey abundance was low (Fig. 1b). Artificial lizards were attacked nearly twice as frequently in the dry season compared to the wet season, corresponding to low and high invertebrate prey abundances, respectively. There was no significant effect of microhabitat (models placed on trees vs. ground; *P* = 0.541) or habitat type (Reid River box vs. Silver-leaf ironbark; *P* = 0.260; Table 2) on predation risk. Our attack rates on model lizards likely represent a conservative estimate because all models that were stepped on (by cattle or humans; *n* = 2) or were missing upon collection (lizard shape: *n* = 41; control shape: *n* = 43) were removed from analysis because the fate of the model could not be determined. We realize some missing models may have been removed by predators, but without retrieving the artificial lizards for inspection, their fate could not be accurately quantified. We acknowledge that our estimate of predation are therefore conservative, but missing models only accounted for 7% of the total models deployed.

**Table 1:** Attack frequencies from artificial lizard models.

		Total	Habitat		Microhabitat		Time			Predator group	
			Box	Ironbark	Ground	Tree	Day	Night	Bird	Invertebrate	Other
Dry season	Lizard shape deployed	400	200	200	200	200	—	—	—	—	—
	Models missing*	10	3	7	10	0	—	—	—	—	—
	Attacked	74	33	41	39	35	—	—	68	5	1
Wet season	Lizard shape deployed	400	200	200	200	200	—	—	—	—	—
	Models missing*	31	18	13	28	3	23	8	—	—	—
	Attacked	43	20	23	20	23	20	23	31	10	2
	Control shape deployed	400	200	200	200	200	—	—	—	—	—
	Models missing*	43	31	12	37	6	31	12	—	—	—
	Attacked	19	11	8	14	5	8	11	9	7	3

Artificial models were placed in two habitat types: Reid River box (*Eucalyptus brownii*; “Box”) and Silver-leaf Ironbark (*Eucalyptus melanophloia*; “Ironbark”) and two microhabitats: on the ground (“Ground”) or on the trunks of trees (“Tree”). Model fate was checked at dawn (“Night”) and dusk (“Day”) to identify predation events that occurred throughout the night and day respectively. *Artificial models that could not be recovered were not used in analyses because the fate of the model could not be identified.

**Table 2:** Results from generalized linear mixed-effects models (GLMM) indicating the terms present in the top model as the best predictor for each response variable.

	Response Variable	Terms in top model	Dist.	Coeff.	Lower	Upper	Z value	R.E. Var	*P* value	Response/Post-hoc comparison
Environmental Models	**Global model GLMM: ~ Season + Habitat Type + Microhabitat + (1|Site)**									
	Attacks	Season	B	−0.575	−0.982	−0.167	2.766	0.0	**0.005**	Wet < Dry
		Habitat Type		0.229	−0.169	0.627	1.126		0.260	NS
		Microhabitat		−0.124	−0.520	0.273	0.610		0.541	NS
	Predatory Birds	Season	P	1.815	1.082	2.704	4.471	0.062	**<0.001**	Dry < Wet
		Habitat		1.747	0.906	2.701	3.928		**<0.001**	Box < Ironbark
		Season x Habitat		−1.486	−2.453	−0.642	−3.270		**<0.001**	Dry.Box < Wet.Box; Dry.Box < Dry.Ironbark; Dry.Box < Wet.Ironbark
	Predatory Invertebrates	Season	P	0.030	−0.811	0.872	0.071	0.207	0.943	NS
		Habitat		0.791	−0.165	1.748	1.622		0.105	NS
		Season x Habitat		−0.830	−1.919	0.258	1.495		0.135	NS
	Lizards	Season	P	−0.349	−0.640	−0.063	−2.387	0.087	**0.017**	Wet < Dry
	Invertebrate Prey	Season	P	0.281	0.039	0.526	2.267	0.014	**0.023**	Dry < Wet
Biological Models	**Global model GLMM: ~ Pred.Birds + Pred.Inverts + Lizards + Invert.Prey + (1|Site)**									
	Attacks	Invert.Prey	B	−0.036	−0.072	0.000	−1.976	0.0	**0.048**	(−)
	Predatory Birds	Lizards	P	−0.034	−0.081	0.012	1.438	0.137	0.150	(−)
	Predatory Invertebrates	Lizards	P	−0.050	−0.120	0.020	1.401	0.048	0.161	(−)
		Invert.Prey		0.041	−0.022	0.103	1.274		0.202	(+)
	Lizards	Invert.Prey	P	−0.054	−0.092	−0.016	−3.313	0.017	**<0.001**	(−)
	Invertebrate Prey	Lizards	P	−0.040	−0.070	−0.015	−2.996	0.006	**0.002**	(−)

Response variables represent attacks on artificial lizards (Attacks), abundance of predatory birds (Predatory Birds), abundance of predatory invertebrates (Predatory Invertebrates), abundance of lizards (Lizards), and abundance of alternative (invertebrate) prey (Invertebrate Prey). The model distribution (Dist.; B = binomial, P = Poisson), regression coefficient (Coeff.), lower and upper confidence limits (Lower and Upper, respectively), and Z and P values are presented for each model parameter, and the variance of the random effect (R.E. Var). Responses/Post-hoc comparison indicate Tukey post-hoc tests (*lsmeans*)^63^ for each categorical factor in the top model for the environmental models, and whether the response variable had a positive (+) or negative (−) response to the factors in the top model for the biological models.

Significant *P*-values are represented in bold.

Predatory birds were responsible for a majority of overall artificial lizard attacks (84.6%), followed by large invertebrates (12.8%), and other (unidentifiable) predators (2.6%). While some of the unidentifiable attacks or missing models could have come from small predatory mammals, this study system has remarkably low numbers of small mammals. Neilly and Schwarzkopf (unpublished data) report low capture rates of small mammals at these sites, with only 39 captures from 20,160 trap nights over 3 years. This trend was similar to other dry savanna woodlands across northeast Queensland^33^. We suspect that most of the bird attacks on artificial lizards came from large predatory birds abundant at our sites, including grey butcherbirds (*Cracticus torquatus*), pied butcherbirds (*Cracticus nigrogularis*), Australian magpies (*Cracticus tibicen*), blue-winged kookaburras (*Dacelo leachii*), laughing kookaburra (*Dacelo novaeguineae*), Torresian crows (*Corvus orru*), and Australian ravens (*Corvus coronoides*). We found no significant difference in the rate of attacks on artificial lizards from birds during the day (58%) or night (42%) (*t* = 0.478, df = 11, *P* = 0.641), likely because many of these birds are crepuscular foragers. Further, we found no difference in the overall attack rates on artificial lizards during the day (47%) or night (53%) (*t* = 0.289, df = 13, *P* = 0.776). Invertebrate predators were likely huntsman spiders (Sparassidae) and centipedes (Scolopendromorpha) based on bite mark indentations left on artificial lizards and the high abundance of these invertebrates throughout the study area. Most of the attacks by invertebrates occurred at night (90%). We did not detect any attacks on artificial lizards from snakes or other reptiles. Predation risk was not correlated to the abundance of either predator group (birds: *rho* = −0.017, *P* = 0.949; invertebrates: *rho* = 0.049, *P* = 0.854).

### Lizard and predator abundance

Our top models (ΔAICc < 2) indicated that *invertebrate prey abundance* and *season* were the best predictor of gecko abundance (Table 2). Lizards were more abundant in the dry season than the wet season, a trend opposite to invertebrate prey abundance (Fig. 1b, Table 3). We conducted 13.3 hrs of dawn point-count surveys for diurnal predatory birds, and 13.3 hrs of nocturnal spotlight surveys for nocturnal predators, including large invertebrates, nocturnal birds, and snakes. We identified 14 species of predatory birds, two groups of predatory invertebrates, and one species of snake (Table 4). Magpies, butcherbirds, and corvids made up 71.8% of all the predatory birds detected. Pale-headed snakes (*Hoplochephalus bitorquatus*) were the only snakes we encountered, but are nocturnal, arboreal, and a likely a predator of small lizards^34^. Huntsman spiders were the most abundant invertebrate predator and made up 73.7% of the invertebrate predator abundance.

**Table 3:** Mean (±SE) abundance counts for *Gehyra dubia* (calculated from a combination of nocturnal spotlighting surveys and captures under artificial cover boards (ACBs)) and invertebrate prey abundance (calculated from under ACB surveys).

		Total	Habitat	
		Abundance	Box	Ironbark
Dry season	*Gehyra dubia*	12.2 ± 0.71	12.5 ± 0.80	11.5 ± 1.46
	Invertebrate prey	2.9 ± 0.36	2.9 ± 0.57	2.8 ± 0.46
Wet season	*Gehyra dubia*	8.7 ± 0.88	9.0 ± 1.16	7.6 ± 1.28
	Invertebrate prey	3.8 ± 0.25	3.7 ± 0.34	1.7 ± 0.39

Data summarized from all eight sites (Total) or 4 sites for each habitat type. Note that gecko abundance was surveyed and summarized over each 1 ha. site (through active spotlighting and the use of ACBs), whereas invertebrate prey abundance was surveyed and summarized based on area-defined surveys defined under ACBs (24 ACBs per site at 0.25 m^2^ each; 1 site represents a total search area of 6 m^2^).

**Table 4:** Total counts of predator groups.

Predator Group	Species	Count	Dry Season		Wet Season		
			Habitat			Habitat	
			RRB	SLI	Count	RRB	SLI
Birds	Blue-faced Honey-eater (*Entomyzon cyanotis*)	3	3	0	8	6	2
	Brown Goshawk (*Accipiter fasciatus*)	0	0	0	1	0	1
	Corvids (*Corvus sp*.)	11	0	11	23	5	18
	Grey Butcherbird (*Cracticus torquatus*)	4	1	3	0	0	0
	Pied Butcherbird (*Cracticus nigrogularis*)	6	0	6	23	17	6
	Grey-crowned Babbler (*Pomatostomus temporalis*)	9	1	8	0	0	0
	Kookaburra (*Dacelo sp*.)	3	1	2	7	4	3
	Australian Magpie (*Cracticus tibicen*)	6	0	6	31	7	24
	Pheasant Coucal (*Centropus phasianinus*)	2	1	1	0	0	0
	Southern Boobook Owl (*Ninox boobook*)	0	0	0	1	1	0
	Tawny Frogmouth (*Podargus strigoides*)	0	0	0	2	2	0
	Whistling Kite (*Haliastur sphenurus*)	4	0	4	4	1	3
Invertebrates	Centipedes (*Scolopendra sp*.)	9	7	2	19	19	0
	Huntsman spiders (*Sparassidae*)	67	30	37	81	45	36
	Redback Spider (*Latrodectus hasseltii*)	11	4	7	1	1	0
Snakes	Pale-headed Snake (*Hoplocephalus bitorquatus*)	0	0	0	2	2	0

RRB = Reid River box; SLI = Silver-leaf ironbark.

### Predatory bird abundance

**Figure 2:**
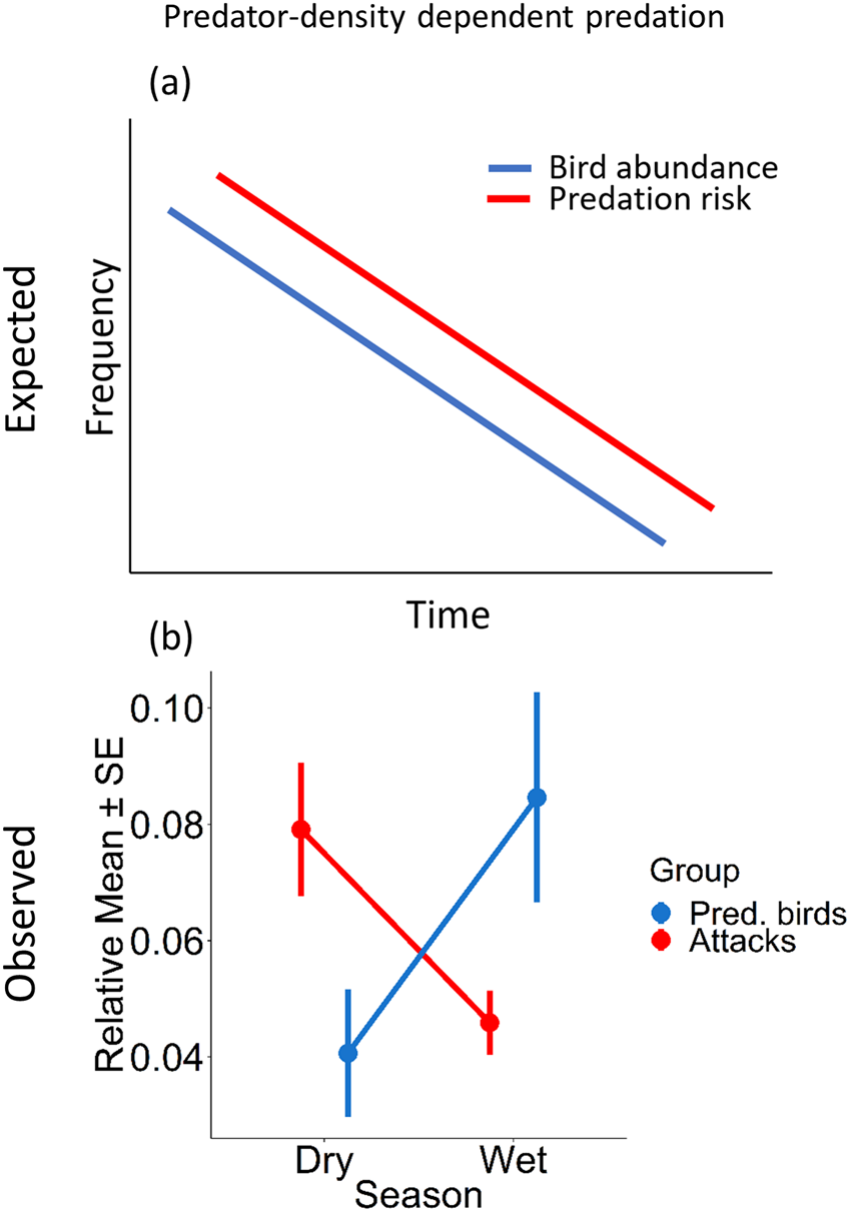
Expected (**a**) and observed (**b**) results for the predator density-dependent predation hypothesis, i.e., predation risk increases with an increase in predator abundance (blue line; **a**). The observed values for the predator density-dependent predation risk (**b**) indicate that predation risk was not predator density-dependent, as the relative mean number of attacks on lizard models (Attacks; red) and the relative mean abundance of predatory birds (Pred. Birds; blue) show an inverse relationship to each other. The relative means represents a proportional response of attacks on artificial lizard models and predatory bird abundance summarized by season to scale the data from 0–1 for direct comparisons of trends.

Our top models for the best predictors of predatory bird abundance included a *season* x *habitat* interaction and a non-significant negative relationship with *gecko abundance* (Table 2). Predatory birds were more abundant in the wet season than the dry season, and in the ironbark than the box habitat (Fig. 2; Table 4).

### Snake and Invertebrate predators

Snake abundances were too low throughout the duration of this study to adequately produce models using snake abundance, although they are likely potential predators (see Appendix S1 in Supporting Information). We only found two snakes, both pale-headed snakes (*Hoplocephalus bitorquatus*) in box habitat in the wet season. Invertebrates (predominantly huntsman spiders) were responsible for 13% of attacks on artificial lizards. Large predatory invertebrates may be important predators of small vertebrates, such as lizards, even though invertebrates are often overlooked in this capacity^35^ (see Appendix S1). A *season* x *habitat* interaction was the best predictor of predatory invertebrate abundance (Table 2). There was a trend for the ironbark habitat to support higher abundances of predatory invertebrates than the box habitat in both the dry and wet season (see Fig. S1).

### Invertebrate prey

Our top models included *season* and *gecko abundance* as the best predictors of invertebrate prey abundance (Table 2). Invertebrate prey abundance was highest in the wet season and lowest in the dry season (Fig. 1b) and were significantly negatively related to gecko abundance (*P* = 0.002).

## Discussion

In our study, predation risk on lizards was not proportional to the abundance of predatory birds, lending no support to the hypothesis that predator density drives predation risk. Instead, we found an inverse relationship between predator abundance and predation risk, with higher predation rates at low bird abundances. We found the abundance of alternative prey (invertebrates), was also inversely related to lizard predation; i.e., when the abundance of alternative prey was low, predation risk was greater for lizards, lending strong support to the alternative prey hypothesis. Predation risk on small lizards oscillated over time in opposition to the rise and fall of invertebrate prey populations, consistent with the alternative prey hypothesis, and opposing the predator density-dependence hypothesis. Our study constitutes one of the few empirical studies of the factors driving predation risk, directly, rather than inferring predation risk from indirect measures (such as predator presence or behavioural changes in prey behaviour).

Few studies present data on predator abundances and densities when discussing predator–prey interactions, although predator abundance and density may be major contributing factors for both consumptive and non-consumptive effects on prey populations. Liebezeit and Zack (2008) monitored nests of ground-dwelling birds in the Arctic using remote cameras, and identified potential nest predators from timed point-count surveys. They found more avian predators in the environment (80%), yet Arctic foxes were responsible for over 80% of the predation events captured on remote cameras. Although there were high densities of avian predators, there was a weak association between predation risk and avian predation on nests^36^. Similarly, high density of predators does not necessarily mean they are responsible for increased predation. For example, DeGregorio *et al*.^37^ found high predation of passerine bird nests near powerlines and at habitat edges where raptors were abundant, yet video surveillance of nests indicated that snakes were responsible for the majority of nest predation events. Predator groups can often be misrepresented without confirmation of predation attempts from remote cameras^36,37^, artificial/clay models (e.g. teeth indentations^11,38^, or visual confirmation. Without confirmation of predation events, abundant ‘potential’ predators may be erroneously classified as important predators in an ecosystem, inferred directly from their abundance^37^.

Birds are the most common predators of small herpetofauna in many ecosystems (frogs^11,39,40^; lizards^39,41^; snakes^38,42^). We identified predator groups from indentations on artificial lizards after predation attempts, enabling us to conclude predatory birds were responsible for a majority of attacks on artificial lizards (84.6%). Both grey (*Cracticus torquatus*) and pied butcherbirds (*Cracticus nigrogularis*) were common in our study area (21.2% of total bird abundance) and often foraged around and under loose or peeling bark^43^. Butcherbirds consume *G. dubia*, which are vulnerable while basking in early morning and late afternoon^44^, but may also be dislodged from diurnal refugia under loose or peeling bark. We observed predation by butcherbirds as two geckos fitted with radio transmitters were predated and impaled on dead branches during another study^44^, which is a common behaviour for butcherbirds. Despite having little overlap in activity and foraging time with our nocturnal model species (*G. dubia*), diurnal birds were a major contributor to the predation on our models, especially at dawn and dusk, when many birds were foraging and lizards theromoregulating.

Many of our predatory bird species were generalists and opportunistic feeders^43,45^. In regions where food availability fluctuates seasonally, it is advantageous to consume abundant and easy-to-obtain resources. We suspect that birds in our study alter their diet and foraging strategy to take advantage of the most abundant food sources. Our results indicate a strong seasonal shift in the attack rates on lizards. In the dry season, lizard abundances were the highest, as were predation rates, indicating a positive correlation between predation risk and prey abundance.

The presence of alternative prey (invertebrates) helps explain the fluctuation in predation risk on lizards. When examining the trends in an alternative food source (invertebrates) for predatory birds, we found that they followed the opposite pattern from lizard abundance; invertebrates were most abundant in the wet season, and least abundant in the dry season. The pattern in predator and alternative prey abundances can best be explained by the alternative prey hypothesis, in which predation rates are reduced on lizards when the abundance of alternative prey (invertebrates) increases. We suggest predators took advantage of the most abundant prey source, and switched to an alternative prey when lizard prey populations were depleted. Our estimations of predation risk (highest in the dry season and lowest in the wet season) were consistent with this hypothesis. Similarly, other studies have found greater frequency of vertebrates (lizards and frogs) in the diet of birds during the dry season^39^ and reduced invertebrate abundances during the driest parts of the year^46–49^, although none have also examined predation rates.

The community dynamics in our system of small lizards, predatory birds, and invertebrates make for an interesting predator-prey dynamic. *Gehyra dubia* are an insectivorous lizard, preying on a variety of arthropod prey items, including spiders^50^, but large predatory huntsman spiders have also been known to be predators of juvenile and even adult lizards and frogs^35^. Huntsman spiders would be potential prey items for geckos when they are small, competitors when they are equivalent size, and predators when the spiders grow larger than the geckos. One of the major complexities of this system is in how lizards and spiders are both predators, prey, and competitors of each other. High predation rates on invertebrates by geckos or predatory spiders, may indirectly influence prey-switching in birds.

Many bird species exhibit seasonal shifts in prey preference^13,51,52^. American dippers (*Cinclus mexicanus*) exhibit a seasonal diet shift with a greater proportion of fish in their diet than aquatic invertebrates prior to egg laying^53^. A diet of fish has more calories, lipids, and protein, essential for eggshell formation^53^. The major predatory birds in our study all breed and lay eggs in the dry season^54^. Predatory birds switched from invertebrate to lizard prey based on their relative abundance, but additionally, we hypothesize birds may switch from primarily invertebrate prey to a larger proportion of vertebrates (lizards) during the dry season to acquire additional nutrients prior to breeding and egg laying, although this should be experimentally tested.

We did not find a difference in the attack rates on artificial lizards in relation to microhabitat, although Steffen (2009) found that artificial lizards in the canopy of Costa Rican tropical forests were attacked more frequently than those on the trunk-ground level. We did not examine height differences on the same scale as Steffen (2009); 10 m = canopy vs. 0.5 m = trunk/ground; whereas our height differences were 0 m = ground vs. 2 m = trunk. A majority of the predatory birds in our study foraged on or near the ground^43,45^, which may explain why we didn’t find a difference in the attack rates among the microhabitat types. Although, various factors including geographic location, species composition, and foraging behaviors can lead to dubious comparisons between studies.

In summary, predator–prey dynamics are among the more complicated ecological processes studied by ecologists. Understanding the relationships between predators and prey are even more complex in multi-prey systems. We used a multi-level (bird–lizard–invertebrate) system, and a mensurative experiment to test common, but largely untested, assumptions about the relationship between predator abundance and predation risk. We demonstrated that predator density of the primary predator, birds, was not driving predation risk, and found no support for predator density-dependent predation risk. Abundances of both predators and prey varied between seasons, and we found that predation rates on lizards were greatest in the dry season, at the time we observed the lowest abundances of predatory birds. Thus, although predation risk is often thought to be driven by the number of predators, our results indicated that the relative abundances among multiple prey groups may be more important in determining predation. Predation risk fluctuated inversely with the abundance of alternative prey, suggesting that predation risk was closely tied to alternative prey populations. Predation risk varied seasonally, probably as predatory birds shifted their diet in response to the abundance of alternative (invertebrate) prey, and potentially in relation to nutritional requirements prior to egg laying. Our study directly quantified predation risk in the context of predator–prey dynamics, and determined that in this system, a likely source of increased predation was reduced availability of alternative prey groups, not the abundance of predators. It is difficult to experimentally manipulate the relative abundances of predators and of several prey species on a landscape scale, therefore, experimental tests of the factors driving predation risk could use enclosures stocked with varying densities of predators and prey, or attack rates on artificial models to further test our conclusions.

## Methods

### Study system and site

This study was conducted on at Wambiana Station, a cattle grazing property southwest of Charters Towers, Queensland, Australia (−20.542790, 146.132204, datum = WGS84). The study area is a tropical savanna woodland, containing two major open eucalyptus forest types: Reid River box (*Eucalyptus brownii*) and Silver-leaf ironbark (*Eucalyptus melanophloia*). A total of eight sites (1 ha. each) were distributed across both habitat types, four in each. Sites were 1.52 ± 0.12 km (mean ± SE) apart and were not spatially autocorrelated (Mantel permutation test; *r* = −0.134, *P* = 0.735). We sampled all sites for one-week in the dry season (August 2015) and the wet season (January 2016). During each one-week sampling period, we quantified lizard, predator, and alternative prey abundances, and placed the artificial lizard models to minimize short-term temporal fluctuations in abundance or prey preference.

In this study, we use an empirical, multi-level food web system (birds, lizards, and invertebrates) and a mensurative experiment (seasonal fluctuations in abundance) to test two competing hypotheses regarding predation risk. We selected a locally abundant lizard, the Australian native house gecko (*Gehyra dubia*) as our model to measure predation risk in relation to their predators and the abundance of alternative prey (invertebrates). Native house geckos are small arboreal, insectivorous geckos found throughout eastern Australia^34,50^. While *G. dubia* are primarily nocturnal, they thermoregulate (bask) in the late evening and early morning sun^44^, making them susceptible to predation by many crepuscular predators.

### Lizard model construction

**Figure 3:**
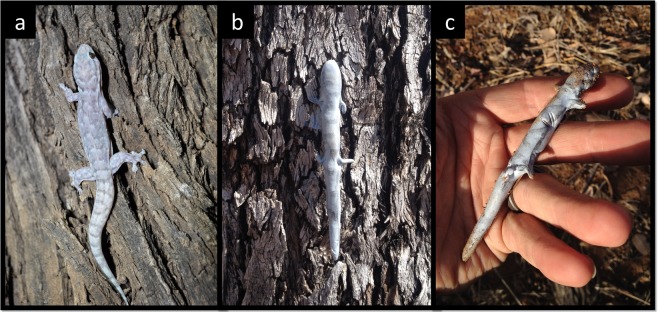
The native house gecko, *Gehyra dubia* (**a**), is an arboreal, nocturnal gecko found throughout northeast Australia. We used Blu-Tack to make physical models of *G. dubia* for deployment in various macro- and microhabitats to test predation risk in lizards (**b**,**c**). Due to its pliable nature, attacks on models can be inferred from indentations remaining after predation events (**c**; indentations from a bird beak). All photographs taken by Eric Nordberg.

We constructed life-sized artificial lizard models (hereinafter as “artificial lizards”) to simulate attack rates and predation attempts on lizards. Plasticine and clay models of small vertebrates, especially herpetofauna, have been widely used to assess predation rates across various habitat types^41,55^, morphological traits such as color pattern^11,40,56^, and body sizes^57,58^. Artificial lizards (Fig. 3) were formed with Blu-Tack adhesive putty (Blu-Tack, Bostik Australia Pty Ltd., Thomastown, VIC, Australia) and were shaped by hand to form lizards with similar dimensions (model snout-vent length = 40–60 mm; mass = 4–6 g) as *Gehyra dubia* (snout-vent length = 47.8 ± 0.65 mm; mass = 3.0 ± 0.10 g; mean ± SE). Prior to shaping artificial lizards, small amounts of graphite powder (Pressol Graphite, Hordern and Company Pty Ltd, Artarmon, NSW, Australia) were added and worked into the Blu-Tack by hand to make models more life-like in coloration. We compared spectral reflectance of artificial lizards and the skin of *G. dubia* using a spectrophotometer to verify that our models produced similar reflectance as actual lizards (Nordberg and Schwarzkopf, unpublished data). We used a graphite pencil to create darker dorsal patterns commonly found in our population of geckos to make lizard models more realistic in appearance. Once the artificial lizards were formed, we placed each model on a transparent plastic sheet cut into lizard shapes (Lowell Laminating Pouches, Officeworks Ltd, Bentleigh, VIC, Australia) with an exposed tab for attaching it to a substrate. A small 20 mm tack was used to secure the model to different microhabitats (e.g., trunks of trees, or the ground).

Due to the pliable nature of Blu-Tack, predation attempts left indentations on the artificial lizards (Fig. 3c). We identified attacking predators by inspecting indentations on the models, and classified predators into categories (e.g., bird, reptile, invertebrate, etc.). The indentations from birds left a deep “V” shape in the model, which could be distinguished from large invertebrates which left two fang or pincer punctures. We validated the indentations created by birds from visual observations of attacks on models. We presented artificial lizards to large huntsman spiders (Sparassidae) and coerced them to bite a lizard model to validate the bite mark indentations from large predatory invertebrates.

### Lizard model placement

In the dry season (August 2015) we placed 400 artificial lizards among our eight sites. We placed models in two microhabitats used by lizards, on the trunks of trees and on the ground. At each site, 25 artificial lizards were placed on the trunks of trees approximately 2 m from the ground and 25 artificial lizards were placed on the ground on various substrata (e.g. bare ground, in leaf litter, under *Carissa ovata* bushes, or on woody debris). Artificial lizards were collected after six days and scored as “not attacked” or “attacked”. Models were only scored as “attacked” if they had bite-mark indentations from a potential lethal predator; bite marks from ants and other small invertebrates were not scored as a potentially lethal attack. Attacked artificial lizards were categorized by predator type. In the wet season (January 2016) we implemented the same procedure for model placement but with the addition of 400 control models (patternless Blu-Tack rolled by hand into spheres) to test if predators could distinguish foreign objects from lizard-shaped models. We hypothesized that if control models were attacked at the same rate as artificial lizards, then attack rates were possibly a result of predators inspecting novel objects and may not accurately depict predation. Further, in the wet season, we checked the fate of models twice daily, just after dawn to check for nocturnal attacks, and just before sunset to check for diurnal attacks to test for differences in attack rates from diurnal vs. nocturnal predators.

### Invertebrate predator and prey abundance

Invertebrate predator and prey abundances were monitored using arboreal cover boards (ACBs)^59^. Closed-cell foam cover boards (50 × 50 × 1 cm) were strapped to the main trunks of trees using elastic bungee cords approximately 1.5 m from the ground. Cover boards were used as area-constrained surveys to quantify the relative abundance of invertebrate prey between seasons. All cover boards were removed every day during morning surveys (between 0800–1000 hrs) to quantify the abundance of invertebrate prey but also to survey for sheltering lizards. Cover boards remained on a particular tree for two consecutive days before being moved and replaced on different trees throughout each site. Twenty-four trees were sampled at each site, for a total of 192 trees or 384 trap-nights. During each season, relative invertebrate prey abundances were calculated from area-constrained searches (each ACB represents 0.25 m^2^ (50 cm × 50 cm) multiplied by 24 trees = 6 m^2^ of search area per site). This method has been used to monitor small invertebrate prey groups, such as beetles, ants, crickets, and spiders, as well as large invertebrate predators such as centipedes and huntsman spiders^50,59^.

### Nocturnal surveys: geckos and predator abundance

Gecko abundances were monitored via timed spotlight surveys and the use of arboreal cover boards (see description above). Nocturnal surveys were conducted every night, consisting of time-constrained spotlight surveys for geckos and nocturnal predators (i.e., birds, snakes, invertebrates). Each spotlight survey consisted of two observers searching all trees, logs, and the forest floor for 20 min in a “U” shaped transect (to avoid overlap in search area) within each 1 ha. site. All spotlight surveys were completed within the first 3 hours after sunset.

### Diurnal surveys: predatory birds

Diurnal predators (mainly carnivorous or omnivorous birds) were monitored using timed point-count dawn bird surveys. Each morning, in both seasons, a survey was conducted by two observers for 10 min between 0530–0700 hrs. We recorded all birds seen or heard during each survey, and later removed any non-predatory birds (i.e., birds that are not known to consume small lizards^43,45^ from the data).

### Data analysis

We used generalized linear mixed-models (GLMMs; R package *lme4*)^60^ and model selection to identify the best predictor variables for a series of response variables: predation/attack rate, predatory bird abundance, predatory invertebrate abundance, invertebrate prey abundance, and lizard abundance. We constructed a correlation matrix to identify and remove any variables that showed collinearity. Prior to fitting models, we explored variables for collinearity, checked normality of residuals, heterogeneity of variance, and checked our model distribution to avoid overdispersion^61^. Two sets of models were used, “environmental models” used *season*, *habitat type*, and *microhabitat* as predictor variables to our response variables listed above, while our “biological models” used the abundance of other organisms as predictor variables (*predatory bird abundance, predatory invertebrate abundance, lizard abundance*, and *invertebrate prey abundance*; Table 2). All models included *site* as a random factor. Attack frequencies on artificial lizards were analysed using a binomial distribution due to the nature of the response variable (model fate: attacked or not attacked). In all other models dealing with count data we used a Poisson distribution. We conducted model selection with the function ‘dredge’ in the R package *MuMIn*^62^ using the Akaike Information Criterion to identify optimal models (with a ΔAICc < 2). Model averaging was used when no optimal model could be identified (i.e., there were multiple top models with ΔAICc < 2). Final models were validated by examining deviance residual plots. TukeyHSD tests using *lsmeans*^63^ were conducted for post-hoc comparisons. To test differences in attack frequencies between artificial lizards and control (sphere-shaped) models we used a Student’s *t*-test. All analyses were conducted using the program R^64^. All experimental protocols were approved by James Cook University under animal ethics approval A2050 and fieldwork was conducted under the Queensland Department of Environment and Heritage Protection research permit WISP14656614 and were performed in accordance with the relevant guidelines and regulations.

## Supplementary information

Appendix SA1

## Data Availability

Data will be made available from the Dryad Digital Repository.

## References

[1] Lima SL (1998). Nonlethal effects in the ecology of predator-prey interactions. Bioscience.

[2] Chesson P (1978). Predator-prey theory and variability. Annu. Rev. Ecol. Evol. Syst..

[3] Abrams PA (2000). The evolution of predator-prey interactions: theory and evidence. Annu. Rev. Ecol. Syst..

[4] Werner EE, Gilliam JF, Hall DJ, Mittelbach GG (1983). An experimental test of the effects of predation risk on habitat use in fish. Ecology.

[5] Schmitz OJ, Beckerman AP, O’Brien KM (1997). Behaviorally mediated trophic cascades: effects of predation risk on food web interactions. Ecology.

[6] Schmitz OJ (1998). Direct and indirect effects of predation and predation risk in old‐field interaction webs. Am. Nat..

[7] Heithaus MR, Dill. LM (2002). Food availability and tiger shark predatin risk influence bottlenose dolphin habitat use. Ecology.

[8] Valeix M (2009). Behavioural adjustments of Arfican herbivores to predation risk by lions: spatiotemporal variations influence habitat use. Ecology.

[9] Brodie EDI (1993). Differential avoidance of coral snake banded patterns by free-ranging avian predators in Costa Rica. Evolution (N. Y)..

[10] Marini MA, Robinson SK, Heske EJ (1995). Edge effects on nest predation in the Shawnee National Forest, Southern Illinois. Biol. Conserv..

[11] Stuart-Fox DM, Moussalli A, Marshall NJ, Owens IPF (2003). Conspicuous males suffer higher predation risk: visual modelling and experimental evidence from lizards. Anim. Behav..

[12] Norrdahl K, Korpimaki E (2000). Do predators limit the abundance of alternative prey? Experiments with vole-eating avian and mammalian predator. Oikos.

[13] Reif V, Tornberg R, Jungell S, Korpimaki E (2001). Diet variation of common buzzards in Finland supports the alternative prey hypothesis. Ecography (Cop.)..

[14] Iles DT (2013). Predators, alternative prey and climate influence annual breeding success of a long-lived sea duck. J. Anim. Ecol..

[15] Murdoch WW (1969). Switching in general predators: experiments on predator specificity and stability of prey populations. Ecol. Monogr..

[16] Cornell H (1976). Search strategies and the adaptive significance of switching in some general predators. Am. Nat..

[17] Stephens, D. W. & Krebs, J. R. *Foraging theory*. (Princeton University Press, 1986).

[18] Hughes RD, Croy MI (1993). An experimental analysis of frequency-dependent predation (switching) in the 15-spined stickleback, *Spinachia spinachia*. J. Anim. Ecol..

[19] Abrams PA, Matsuda H (1996). Positive indirect effects between prey species that share predators. Ecology.

[20] Abrams PA, Matsuda H (2003). Population dynamical consequences of reduced predator switching at low total prey densities. Popul. Ecol..

[21] Holt RD, Lawton JH (1994). The ecological consequences of shared natural enemies. Annu. Rev. Ecol. Syst..

[22] Lack, D. *The natural regulation of animal numbers*. (Oxford University Press, 1954).

[23] Hörnfeldt, B. Synchronous population fluctuations in voles, small game, owls, and tularemia in northern Sweden. *Oecologia*, 10.1007/BF00366068 (1978) 10.1007/BF00366068 28309394

[24] Gilg O, Hanski I, Sittler B (2003). Cyclic dynamics in a simple vertebrate predator-prey community. Science.

[25] Garrott RA, Bruggeman JE, Becker MS, Kalinowski ST, White PJ (2007). Evaluating prey switching in wolf on ungulate systems. Ecol. Appl..

[26] Essington TE, Hansson S (2004). Predator-dependent functional responses and interaction strengths in a natural food web. Can. J. Fish. Aquat. Sci..

[27] Schmitt RJ, Holbrook SJ (2007). The scale and cause of spatial heterogeneity in strength of temporal density dependence. Ecology.

[28] White JW (2007). Spatially correlated recruitment of a marine predator and its prey shapes the large-scale pattern of density-dependent prey mortality. Ecol. Lett..

[29] White, J. W. & Samhouri, J. F. Oceanographic coupling across three trophic levels shapes source – sink dynamics in marine metacommunities. *Oikos* 1151–1164, 10.1111/j.1600-0706.2010.19226.x (2011).

[30] Miller DA, Grand JB, Fondell TF, Anthony M (2006). Predator functional response and prey survival: direct and indirect interactions affecting a marked prey population. J. Anim. Ecol..

[31] Hollander FA, Van Dyck H, Martin GS, Titeux N (2015). Nest predation deviates from nest predator abundance in an ecologically trapped bird. PLoS One.

[32] McKinnon L, Berteaux D, Bêty J (2014). Predator-mediated interactions between lemmings and shorebirds: a test of the alternative prey hypothesis. Auk.

[33] Kutt AS, Gordon IJ (2012). Variation in terrestrial mammal abundance on pastoral and conservation land tenures in north-eastern Australian tropical savannas. Anim. Conserv..

[34] Wilson, S. K. *A field guide to reptiles of Queensland*. (New Holland, 2015).

[35] Nordberg EJ, Edwards L, Schwarzkopf L (2018). Terrestrial invertebrates: an underestimated predator guild for small vertebrate groups. Food Webs.

[36] Liebezeit JR, Zack S (2008). Point counts underestimate the importance of arctic foxes as avian nest predators: evidence from remote video cameras in arctic Alaskan oil fields. Arctic.

[37] DeGregorio BA, Weatherhead PJ, Sperry JH (2014). Power lines, roads, and avian nest survival: Effects on predator identity and predation intensity. Ecol. Evol..

[38] Webb JK, Whiting MJ (2005). Why don’t small snakes bask? Juvenile broad-headed snakes trade thermal benefits for safety. Oikos.

[39] Poulin B (2001). Avian predation upon lizards and frogs in a neotropical forest understorey. J. Trop. Ecol..

[40] Saporito RA, Zuercher R, Roberts M, Kenneth G, Donnelly MA (2007). Experimental evidence for aposematism in the Dendrobatid poison frog *Oophaga pumilio*. Copeia.

[41] Steffen JE (2009). Perch-height specific predation on tropical lizard clay models: implications for habitat selection in mainland neotropical lizards. Rev. Biol. Trop..

[42] Wilgers DJ, Horne EA (2007). Spatial variation in predation attempts on artificial snakes in a fire-disturbed tallgrass prairie. Southwest. Nat..

[43] Barker, R. D. & Vestjens, W. J. M. *Food of Australian Birds 1*. *Non-passerines*. (CSIRO Publishing, 1989).

[44] Nordberg, E. J. & Schwarzkopf, L. Heat seekers: A tropical nocturnal lizard uses behavioral thermoregulation to exploit rare microclimates at night. *Journal of Thermal Biology* **82**, 107–114 (2019). 10.1016/j.jtherbio.2019.03.018 31128638

[45] Barker, R. D. & Vestjens, W. J. M. *Food of Australian Birds 2*. *Passerines*. (CSIRO Publishing, 1990).

[46] James CD (1991). Temporal variation in diets and trophic partitioning by coexisting lizards (*Ctenotus*: Scincidae) in central Australia. Oecologia.

[47] Griffiths AD, Christian KA (1996). The effects of fire on the frillneck lizard (*Chlamydosaurus kingii*) in northern Australia. Austral Ecol..

[48] Recher HF, Majer JD, Ganesh S (1996). Seasonality of canopy invertebrate communities in eucalypt forests of eastern and western Australia. Aust. J. Ecol..

[49] Taylor SG (2008). Leaf litter invertebrate assemblages in box-ironbark forest: composition, size and seasonal variation in biomass. Vic. Nat..

[50] Nordberg EJ, Murray P, Alford R, Schwarzkopf L (2018). Abundance, diet and prey selection of arboreal lizards in a grazed tropical woodland. Austral Ecol..

[51] Cooper, R. J. Dietary relationships among insectivorous birds of an eastern deciduous forest. (West Virginia University, 1988).

[52] Korpimaki E, Huhtala K, Sulkava S (1990). Does the year-to-year variation in the diet of eagle and ural owls support the alternative prey hypothesis?. Oikos.

[53] Morrissey CA, Elliott JE, Ormerod SJ (2010). Diet shifts during egg laying: implications for measuring contaminants in bird eggs. Environ. Pollut..

[54] Beruldsen, G. *Australian birds: thier nests and eggs*. (Self-published, 2003).

[55] Shepard DB (2007). Habitat but not body shape affects predator attack frequency on lizard models in the Brazillian Cerrado. Herpetologica.

[56] Castilla AM, Gosa A, Galan P, Perez-Mellado V (1999). Green tails in lizards of the genus *Podarcis*: do they influence the intensity of predation?. Herpetologica.

[57] Bittner TD (2003). Polymorphic clay models of *Thamnophis sirtalis* suggest patterns of avian predation. Ohio J. Sci..

[58] Mitrovich MJ, Cotroneo RA (2006). Use of plasticine replica snakes to elicit antipredator behavior in the california ground squirrel (*Spermophilus beecheyi*). Southwest. Nat..

[59] Nordberg EJ, Schwarzkopf L (2015). Arboreal cover boards: using artificial bark to sample cryptic arboreal lizards. Herpetologica.

[60] Bates, D., Machler, M., Bolker, B. M. & Walker, S. C. Fitting linear mixed-effects models using lme4. *Jounral Stat. Softw*. 1–51, 10.1126/science.1176170 (2014).

[61] Zuur AF, Ieno EN, Elphick CS (2010). A protocol for data exploration to avoid common statistical problems. Methods Ecol. Evol..

[62] Barton, K. MuMIn: Multi-model inference (2016).

[63] Lenth RV (2016). Least-Squares Means: The R Package lsmeans. Jounral Stat. Softw..

[64] R Core Team. R: A language and environment for statistical computing (2017).

