# Supplementary material for: Predation risk is a function of alternative prey availability rather than predator abundance in a tropical savanna woodland ecosystem
Source: Sci Rep. 2019 May 22;9:7718. doi: 10.1038/s41598-019-44159-6 (PMC6531519; doi:10.1038/s41598-019-44159-6)
Supplement: Supplementary file 1 — Appendix SA1 [file 41598_2019_44159_MOESM1_ESM.pdf]

Predation risk is a function of alternative prey availability rather than predator abundance in a tropical savanna woodland ecosystem

Eric J. Nordberg and Lin Schwarzkopf

College of Science and Engineering, James Cook University, Townsville, QLD 4811, Australia

Appendix SA1 – Additional information and discussion of alternative predators.

Invertebrates: Predators, competitors, prey, or all of the above?

The presence of small lizards and large spiders make for an interesting predator–prey dynamic. Many lizards, including *G. dubia*, are insectivorous and consume a wide variety of prey items, including spiders[1]. Although infrequently studied and observed, many large spiders consume small vertebrates, such as lizards and frogs[2,3]. We suspect that due to the high abundance of *G. dubia* and huntsman spiders at our study site, these two groups are not only competitors for invertebrate prey, but also eat each other. Adult *G. dubia* probably feed on juvenile huntsman spiders, while adult huntsman spiders can consume juvenile (and even some adult) geckos (Nordberg pers. obs). The diets of many web-spinning spiders can be determined by examining food remains and body parts left in the web; whereas for active-foraging spiders, such as huntsmen, diet analysis can be much more difficult. The diets of active-foraging spiders may require burrow excavation[4] or modern techniques such as DNA sequencing of stomach or fecal sample[5] to identify prey items. Nevertheless, we have observed large huntsman spiders consuming *G. dubia* (Nordberg pers. obs.). Others have used plasticine models to record invertebrate "attacks"[6,7] providing insight into predation attempts by predatory invertebrates.

Snake predators

Snakes are likely important predators of *G. dubia* and other small herpetofauna. The most common snake species in our study were pale-headed snakes (*Hoplocephalus bitorquatus*), arboreal specialists that probably feed on a variety of small vertebrates[8]. Unfortunately, we could not detect predation attempts on model lizards by snakes because *H. bitorquatus* are ambush predators; not likely to attack a motionless model[9]. Active foraging snakes, such as coachwhips (*Masticophis flagellum*), do attack even sedentary plasticine models of lizards[10]. Brown tree snakes (*Boiga irregularis*) are nocturnal active foraging snakes present at the study site, but were not observed during our survey periods. Likely, our models underestimated attacks by snakes, but the estimates of survival include predation by snakes.

References

1. Nordberg, E. J., Murray, P., Alford, R. & Schwarzkopf, L. Abundance, diet and prey selection of arboreal lizards in a grazed tropical woodland. *Austral Ecol.* **43,** 328–338 (2018).

2. McCormick, S. & Polis, G. A. Arthropods that prey on vertebrates. *Biol. Rev.* **57,** 29–58 (1982).

3. Nordberg, E. J., Edwards, L. & Schwarzkopf, L. Terrestrial invertebrates: an underestimated predator guild for small vertebrate groups. *Food Webs* **15,** (2018).

4. Henschel, J. R. Diet and foraging behaviour of huntsman spiders in the Namib dunes (Araneae: Heteropodidae). *J. Zool. London* **234,** 239–251 (1994).

5. King, R. A., Read, D. S., Traugott, M. & Symondson, W. O. C. Molecular analysis of predation: A review of best practice for DNA-based approaches. *Mol. Ecol.* **17,** 947–963 (2008).

6. Posa, M. R. C., Sodhi, N. S. & Koh, L. P. Predation on artificial nests and caterpillar models across a disturbance gradient in Subic Bay, Philippines. *J. Trop. Ecol.* **23,** 27 (2007).

7. Saporito, R. A., Zuercher, R., Roberts, M., Kenneth, G. & Donnelly, M. A. Experimental evidence for aposematism in the Dendrobatid poison frog *Oophaga pumilio*. *Copeia* **2007,** 1006–1011 (2007).

8. Wilson, S. K. *A field guide to reptiles of Queensland*. (New Holland, 2015).

9. Huey, R. B. & Pianka, E. R. Ecological consequences of foraging mode. *Ecology* **62,** 991–999 (1981).

10. Husak, J. F., Macedonia, J. M., Fox, S. F. & Sauceda, R. C. Predation cost of conspicuous male coloration in collared lizards (*Crotaphytus collaris*): an experimental test using clay-covered model lizards. *Ethology* **112,** 572–580 (2006).

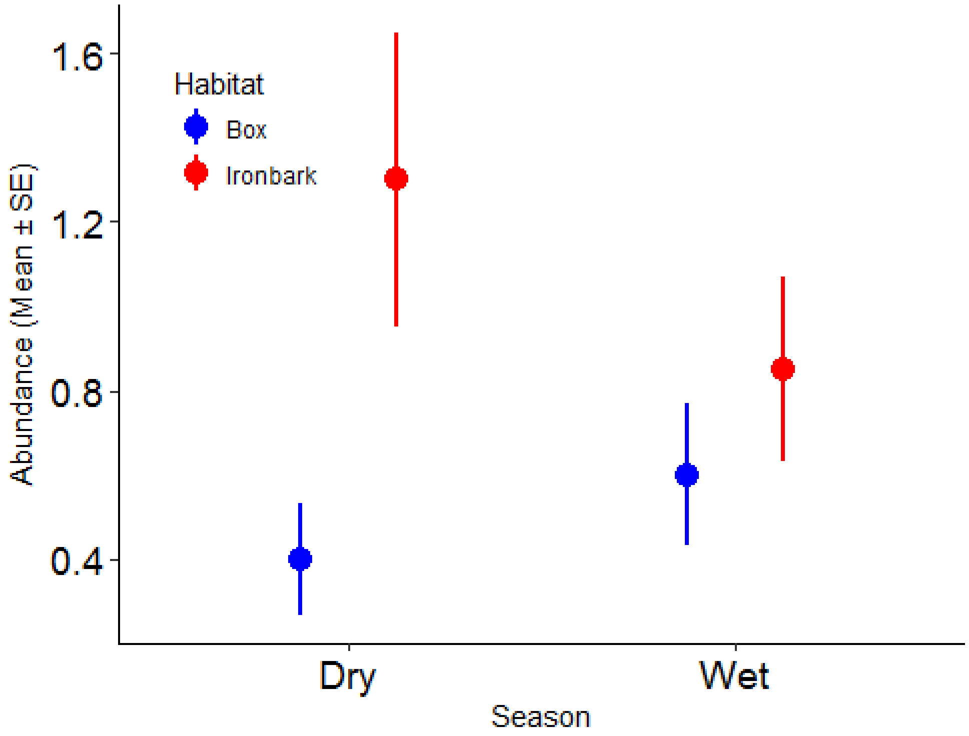

Figure S1. Seasonal and habitat differences in the abundance of predatory invertebrates. Habitat types represent Reid River box (*Eucalyptus brownii*; “Box”; blue) and Silver-leaf ironbark (*Eucalyptus melanophloia*; “Ironbark”; red). Data represents means for each habitat type (four 1-ha sites each) in both the dry and wet season.
